# Foley Catheter for Induction of Labor at Term: An Open-Label, Randomized Controlled Trial

**DOI:** 10.1371/journal.pone.0136856

**Published:** 2015-08-31

**Authors:** Ning Gu, Tong Ru, Zhiqun Wang, Yimin Dai, Mingming Zheng, Biyun Xu, Yali Hu

**Affiliations:** 1 Department of Obstetrics and Gynecology, Nanjing Drum Tower Hospital, Affiliated to Nanjing Medical University, Nanjing, China; 2 Department of Biostatistics, Nanjing Drum Tower Hospital, Affiliated to Nanjing Medical University, Nanjing, China; Oslo University Hospital, Ullevål, NORWAY

## Abstract

**Objective:**

This study aimed to determine the optimal Foley catheter balloon volume (30-mL vs. 80-mL) and the maximum time for cervical ripening (12 hours vs. 24 hours) to improve vaginal delivery rate within 24 hours of induction.

**Methods:**

We conducted an open-label, randomized controlled trial in a teaching hospital in China. Women with a term singleton pregnancy, cephalic presentation, intact membrane and an unfavorable cervix (Bishop score <6) were randomly allocated, in 1:1:1:1 ratio, to receive either one of the four treatments: (1) 30-mL balloon for a maximum of 12 hours, (2) 30-mL balloon for a maximum of 24 hours, (3) 80-mL balloon for a maximum of 12 hours, and (4) 80-mL balloon for a maximum of 24 hours. The primary outcome was vaginal delivery within 24 hours. Secondary outcomes included cesarean section rate and maternal/neonatal morbidity. Data were analyzed on a per-protocol basis.

**Results:**

Five hundred and four women were recruited and randomized (126 women in each group); nine women did not receive the assigned intervention. More women achieved vaginal delivery within 24 hours in 12-hour Foley catheter groups than in the 24-hour Foley catheter groups (30-mL/12 hours: 54.5%, 30-mL/24 hours: 33.1%, 80-mL/12 hours: 46.4%, 80-mL/24 hours: 24.0%, p < 0.001). Cesarean section rates and the incidence of chorioaminonitis were comparable among four groups. After adjustment for confounding factors, both ripening time and balloon size did not affect the proportion of women delivered vaginally within 24 hours of induction.

**Conclusion:**

For women with an unfavorable cervix at term, induction of labor with a Foley catheter is safe and effective. Higher balloon volume (80-mL vs. 30-mL) and longer ripening time (24 hours vs. 12 hours) would not shorten induction to delivery interval or reduce cesarean section rate.

**Trial Registration:**

Chinese Clinical trial registry (ChiCTR-TRC-13003044)

## Introduction

Labor induction is a frequently used method in the management of high-risk pregnancy. At present, both medical and mechanical methods have been applied for cervical ripening in women with an unfavorable cervix. As the oldest methods to induce labor, mechanical methods were developed to promote cervical ripening and the onset of labor by dilating the cervix. Hygroscopic and osmotic dilators are effective, but they might be associated with an increase in maternal infection and are seldom used in the term labor induction [1]. Currently, Foley catheter balloon is the most commonly used mechanical device for labor induction, which acts not only as a mechanical dilator of the cervix but also a stimulator of endogenous prostaglandins release from the fetal membranes.

Double-balloon catheter has been designed and introduced recently for labor induction. However, two studies showed that double-balloon catheter could not improve outcomes and might be associated with more operative deliveries compared with Foley catheter balloon [2,3]. Compared with vaginal prostaglandin E2 gel in term labor induction, Foley catheter achieved similar vaginal delivery rates, with fewer maternal and neonatal side effects [4]. Cost-effectiveness analysis alongside the trial showed that Foley catheter and prostaglandin E2 labor induction resulted in comparable costs [5]. In the Foley catheter group, the induction material was cheaper but induction to delivery interval was longer, which generated higher costs due to longer labor ward occupation.

To improve the efficacy of induction, different balloon inflation sizes and ripening time have been compared. Balloon inflation sizes of 30–80 mL have been reported and two randomized controlled trials showed that larger balloon volume was associated with shorter induction to delivery interval without affecting cesarean section rate [6,7]. As to the time limitation for exposure to extra-amniotic balloon, some practitioners set a maximum time limit [3,6], while others wait until spontaneous expulsion of the balloon catheter [7,8]. Cromi et al [9] reported that shortening the maximum time for cervical ripening (from 24 to 12 hours) might increase the proportion of women who delivered vaginally within 24 hour after Foley catheter insertion.

As the previous studies suggested, both balloon size and ripening time might affect the efficacy of induction; however we had not been able to identify any published data that has explored these two conditions in the same trial. The aim of this study was to determine the optimal balloon volume (30-mL vs. 80-mL) and cervical ripening time (12 hours vs. 24 hours) to improve the proportion of women delivered vaginally within 24 hours of induction.

## Methods

### Subjects and sample size assessment

This study was performed according to the Declaration of Helsinki and approved by the institutional review boards of Nanjing Drum Tower Hospital (Protocol number: 2013004). Each woman gave the written informed consent to participate in this study.

We intended to conduct a prospective, multicenter, open-label, randomized controlled clinical trial in five hospitals of Jiangsu Province, China, and registered with the Clinical trial registry (ChiCTR-TRC-13003044, http://www.chictr.org/en/). However, four other hospitals failed to enroll the participants according to the randomization principle so were excluded from the study. We revised the study protocol in the early stage of the trial and performed a single center trial. The data were collected in Nanjing Drum Tower Hospital, which is a tertiary hospital, between February 2013 and April 2014. Women scheduled for labor induction were screened for eligibility. Inclusion criteria were gestational age ≥ 37 weeks; singleton gestation with cephalic presentation; intact membranes; reactive non-stress test and Bishop’s score < 6. Women with vaginal infection, antepartum bleeding, intrauterine fetal death, placenta previa, or any other contraindication to vaginal delivery were excluded.

To investigate the labor patterns in women induced with Foley catheter versus spontaneous labor, we prospectively collected the information of women with spontaneous onset of labor and vaginal delivery in a 1:1 ratio. Women were eligible as control group if they were nulliparous, singleton pregnancy in vertex presentation, fewer than 2 cm dilated with intact membranes on admission, and spontaneous onset of labor. We excluded women who delivered preterm, had fetuses with congenital anomalies, or delivered by cesarean section. Maternal age was matched and difference in gestational age at delivery should be fewer than 7 days. We extracted detailed information on maternal demographic, obstetric history, antepartum records, and labor and delivery records. The labor and delivery information included epidural analgesia, cervical dilation and station, length of labor stages, mode of delivery, postpartum hemorrhage (PPH), and neonatal admission. For this study, we compared the duration of the latent phase, active phase, first stage of labor and second stage of labor. Women in the spontaneous labor group may have received augmentation with oxytocin or undergone artificial rupture of membranes.

Our previous study using an 80-mL balloon with a maximum of 24 hours indicated a vaginal delivery rate in 24 hours of 28.1% [10]. Studies of various balloon sizes and ripening time reported a vaginal delivery rate within 24 hours ranged from 48% to 66% [2,4,6]. Under this scenario, we assumed that by revising the Foley catheter induction protocol we could achieve a clinically significant increase in vaginal delivery within 24 hours. Thus planned sample size for this investigation was 126 patients each treatment group to show an increase in vaginal delivery rate within 24 hours from 28% to 48% with a two-sided test (α error = 5%, power = 80%).

### Randomization

The randomization sequence was computer-generated with variable blocks in 1:1:1:1 allocation for each arm by the statistician (Dr. Xu). The sequence was placed into numbered opaque envelopes by an uninvolved third party before the initiation of the study. At enrollment, the obstetricians recorded medical history and reviewed the indication for induction and explained the objectives of the study to the pregnant women. Gestational age was calculated by last menstrual period (LMP) and/or first ultrasound scan. Dating was based on LMP if ultrasound’s results were in agreement with that estimated by LMP within 7 days up to 19 6/7 weeks, within 14 days at 20–29 6/7 weeks, or within 21 days at 30 weeks or beyond. If LMP data were not available, dating was based on the first ultrasound scan. The status of the cervix was determined by the Bishop pelvic scoring system [11]. After informed consent was obtained from eligible participants, the obstetricians opened the consecutively numbered envelope and assigned the participants to corresponding groups.

### Interventions

Before the trial began, all the staff had been trained on standard operative protocol and Bishop scoring system. The cervix was exposed with a sterile speculum and cleansed with a povidone-iodine solution. Under direct visualization, a 16-F Foley catheter (bardic Foley Catheter, Bard international, Inc.) was inserted into the endocervical canal. Once the catheter was past the internal os, the balloon was inflated with sterile saline solution and pulled against the internal os of the cervix. The external end of Foley catheter was taped with tension to the medial aspect of the woman’s thigh. Non-stress test was conducted after catheter insertion.

Women in group 1 and group 2 had a 30-mL Foley catheter balloon, with a maximum ripening time of 12 hours and 24 hours respectively. Women in group 3 and group 4 had an 80-mL Foley catheter balloon with a maximum of 12 hours and 24 hours exposure respectively. In this study, we define “ripening time” as the maximum time limit for Foley catheter balloon exposure. In all groups, the Foley catheter was removed for the following reasons: (1) the time limit for ripening was reached; (2) spontaneous rupture of membranes occurred; (3) the balloon was expelled spontaneously; (4) women entered the active phase of labor; or (5) hyperstimulation or fetal distress was suspected.

For women who did not enter spontaneous labor during the ripening process, amniotomy was preformed once the catheter was expelled or removed. Women were given intravenous oxytocin to induce the labor if the contraction frequency was unsatisfactory after 30 minutes of amniotomy (< 3 contractions per 10 min). Intravenous oxytocin was started at an infusion rate of 1 mIU/min. The dose was increased by 2 mIU/min every 20 minutes until adequate uterine activity was achieved, defined as uterine activity of 200–250 Montevideo units. The maximal infusion rate permitted was 25 mIU/min. Oxytocin was discontinued once the women were deemed to be in active labor. When a woman was in labor, maternal heart rate, blood pressure and temperature were recorded every 6 hours.

The criteria for onset of labor were painful contractions accompanied by effacement of at least 80 percent. Active phase was defined as complete cervical effacement and dilatation of at least 4 cm. When the labor was established, fetal heart rate and uterine activity was continuously recorded with fetal heart monitor.

Hyperstimulation was defined as more than 5 uterine contractions in 10 minutes in a consecutive 30-minute interval with or without fetal heart rate changes [12]. Failed induction was identified as failure to progress into the active phase of labor, despite adequate contraction patterns 24 hours after amniotomy. Failure to progress was diagnosed as unchanged cervical dilation in a 4-hour interval, despite the establishment of well uterine contraction. Chorioamnionits was diagnosed as maternal fever (≥38°C), accompanied by maternal tachycardia (>100 bpm), or uterine fundal tenderness, or fetal tachycardia (>160 bpm), or purulent amniotic fluid [13]. PPH was defined as blood loss of more than 500 mL within 24 hours of delivery. Blood loss was measured by: 1) collecting and recording of blood in bedpan containers, and 2) weighing of materials including soaked sponges and pads on a scale and subtracting the known dry weights of these materials.

### Outcomes

The primary outcome was vaginal delivery within 24 hours. Secondary maternal outcomes were cesarean section rate, instrumental vaginal delivery, and indications for operative delivery, induction-to-active phase interval, induction-to-delivery interval, uterine hyperstimulation, chorioamnionitis and PPH. Secondary neonatal outcomes included birth weight, the rate of Apgar scores of fewer than 7 at 5 minutes and neonatal admission.

### Statistical methods

Statistical analysis was performed with SPSS 17.0. Data were analyzed on a per-protocol basis. Normally distributed data were presented as mean with SD; skewed distributions were presented as median with interquartile range (IQR). Categorical outcomes were summarized using frequency distributions. For quantitative data, ANOVA or rank sum test was used. For categorical data, we calculated p values with Chi-square or Fisher exact tests. For time-to-delivery data, we constructed Kaplan-Meier survival curves and calculated log-rank test and p values. In the survival curve analysis for induction to active phase interval, we used “cervical dilation of 4 cm” as the endpoint. To analyze induction to vaginal delivery interval, we used “vaginal delivery” as the endpoint. A multivariable Logistic regression model was used to estimate the adjusted effect of balloon size and ripening time on vaginal delivery within 24 hours. COX multiple regression tests were used to explore variables independently associated with the induction to delivery interval. A p value of 0.05 was used as the cut-point for significance.

## Results

**Fig 1:**
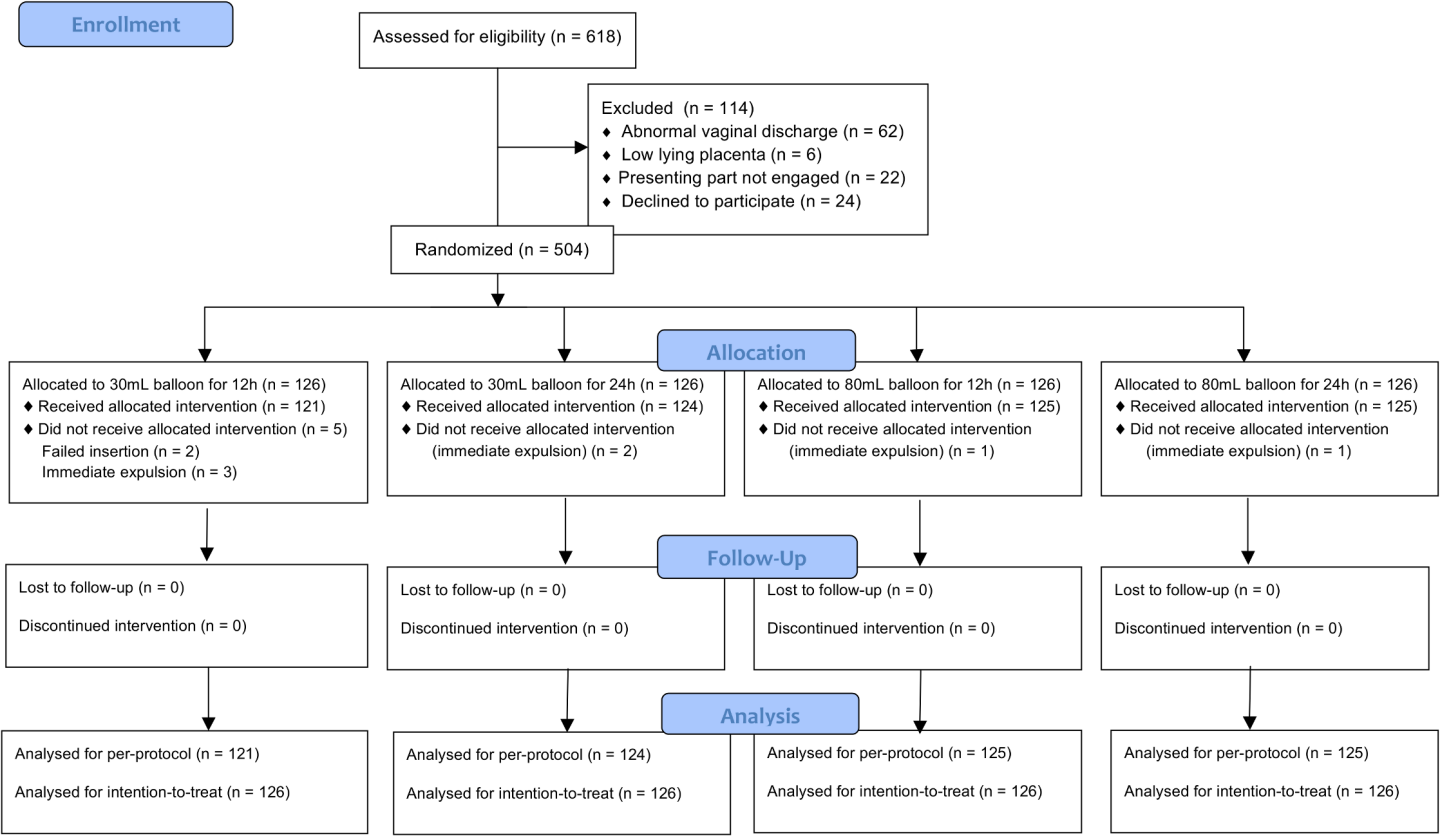
Participants flowchart through the trial.

During the study period, 618 women were screened for eligibility. Flow of participants through the randomized clinical trial is shown in Fig 1. Of them, 24 declined to participate in the trial, 62 and 6 were excluded because of abnormal vaginal discharge and low lying placenta respectively, and 22 were considered inappropriate for Foley catheter insertion because the presenting part was not engaged. Thus, a total of 504 women were randomized in this investigation. However, nine women did not receive the allocated intervention. The Foley catheter placement was failed in 2 women, who later both received PGE2 vaginal inserts for labor induction. The devices were expulsed immediately after insertion in 7 patients. Two of them had oxytocin induction and 5 of them were converted to PGE2 vaginal inserts. These nine women were included in an intention-to-treat analysis.

The baseline characteristics of the participants were comparable between groups (Table 1). Ninety three percent of the women were nulliparous and Bishop scores before cervical ripening were similar among groups. Indications for induction of labor were comparable among groups, with the majority of women undergoing induction for prolonged gestation and gestational or pre-gestational diabetes. The induction indication “other” refers to the combined indications such as maternal nephritis, cholestasis and non-reassuring cardiotocography.

**Table 1:** Baseline characteristics of subjects.

	30-mL/12h (n = 121)	30-mL/24h (n = 124)	80-mL/12h (n = 125)	80-mL/24h (n = 125)	p
Maternal age (years; mean ± SD)	28.0 ± 3.4	28.4 ± 3.5	28.4 ± 3.4	28.4 ± 3.7	0.829
Parity n (%)					0.684
0	114 (94.2)	117 (94.4)	113 (90.4)	118 (94.4)	
1	7 (5.8)	6 (4.8)	11 (8.8)	7 (5.6)	
2	0 (0.0)	1 (0.8)	1 (0.8)	0 (0.0)	
Bishop score (median [IQR])	4 (4–5)	4 (4–5)	4 (4–5)	4(4–5)	0.265
GA (weeks; median [IQR])	40.1 (39.3–41.0)	41.0 (39.4–41.0)	40.0 (39.1–41.0)	40.4 (39.6–41.0)	0.360
Indications for induction n (%)					
GA ≥ 41 weeks	59 (48.8)	73 (58.9)	55 (43.7)	56 (44.8)	
GDM/Diabetes	42 (34.7%)	31 (25.0%)	31 (24.8%)	43 (34.4%)	
HDP	8 (6.6%)	7 (5.6%)	19 (15.2%)	13 (10.4%)	
FGR	1 (0.8%)	2 (1.6%)	8 (6.4%)	3 (2.4%)	
Oligohydramnion	9 (7.4%)	7 (5.6%)	9 (7.2%)	9 (7.2%)	
Other	2 (1.7%)	4 (3.2%)	3 (2.4%)	1 (0.8%)	

GA, gestational age. GDM, gestational diabetes mellitus. HDP, hypertensive disorders in pregnancy. FGR, fetal growth restriction.

More women achieved vaginal delivery within 24 hours in 12-hour Foley catheter groups than in the 24-hour Foley catheter groups (12-hour vs. 24-hour Foley catheter: 50.4% vs. 28.5%, OR 2.548, 95% CI 1.757–3.695). When the maximum ripening time was set to12 hours, vaginal delivery rate within 24 hours was higher in the 30-mL group compared with the 80-mL study arm, although this did not reach statistical significance (30-mL/12h vs. 80-mL/12h Foley catheter: 54.5% vs 46.4%: OR 1.386, 95% CI 0.839–2.289, Table 2). Logistic regression analysis showed that independent factors for vaginal delivery rate within 24 hours included parity, gestational age and neonatal birth weight. Correction for these factors revealed that both ripening time (12-hour vs. 24-hour Foley catheter: OR 2.445, 95% CI 0.733–8.610) and balloon size (30-mL vs. 80-mL Foley catheter: OR 1.326, 95% CI 0.405–4.342) did not affect the proportion of women delivered vaginally within 24 hours of induction.

**Table 2:** Induction to delivery interval and mode of delivery.

	30-mL/12h (n = 121)	30-mL/24h (n = 124)	80-mL/12h (n = 125)	80-mL/24h (n = 125)	p
Vaginal delivery within 24 hours (h) n (%)	66(54.5)	41(33.1)	58(46.4)	30(24.0)	< 0.001
Time to delivery (h) (median [IQR])	22.9 (19.5–33.0)	29.2 (20.6–41.2)	23.8 (18.0–30.5)	29.5 (24.2–37.7)	< 0.001
Time to active phase (h) (median [IQR])*	19.0 (16.5–23.0)	22.7 (14.5–31.3)	19.0 (15.3–23.3)	25.5 (17.2–31.3)	< 0.001
Time to vaginal delivery (h) (median [IQR])*	22.5 (19.1–27.7)	25.6 (18.2–34.8)	21.5 (17.3–28.5)	28.8 (21.6–35.9)	0.001
Mode of delivery n (%)					0.621
Spontaneous	104 (86.0)	95 (76.6)	98 (78.4)	98 (78.4)	
Vaginal instrumental	3 (2.4)	3 (2.4)	3 (2.4)	3 (2.4)	
Cesarean section	14 (11.6)	26 (21.0)	24 (19.2)	24 (19.2)	
Indications for Cesarean section n (%)					0.514
Failed induction	7 (50.0)	10 (38.5)	6 (25.0)	12 (50.0)	
Failure to progress	5 (35.7)	11 (42.3)	15 (62.5)	10 (41.6)	
Fetal distress	1 (7.1)	2 (7.7)	0 (0.0)	0 (0.0)	
Chorioamionitis	1 (7.1)	3 (11.5)	3 (12.5)	2 (8.3)	
Indications for vaginal instrumental delivery n (%)					0.721
Failure to progress	3 (100.0)	2 (66.7)	2 (66.7)	2 (66.7)	
Fetal distress	0 (0.0)	1 (33.3)	1 (33.3)	1(33.3)	
Oxytocin use n (%)	106 (87.6)	109 (87.9)	108 (86.4)	113 (90.4)	0.800
Epidural analgesia n (%)	24 (19.8)	26 (21.0)	15 (12.0)	23 (18.4)	0.251

* Excluding cesarean deliveries.

**Fig 2:**
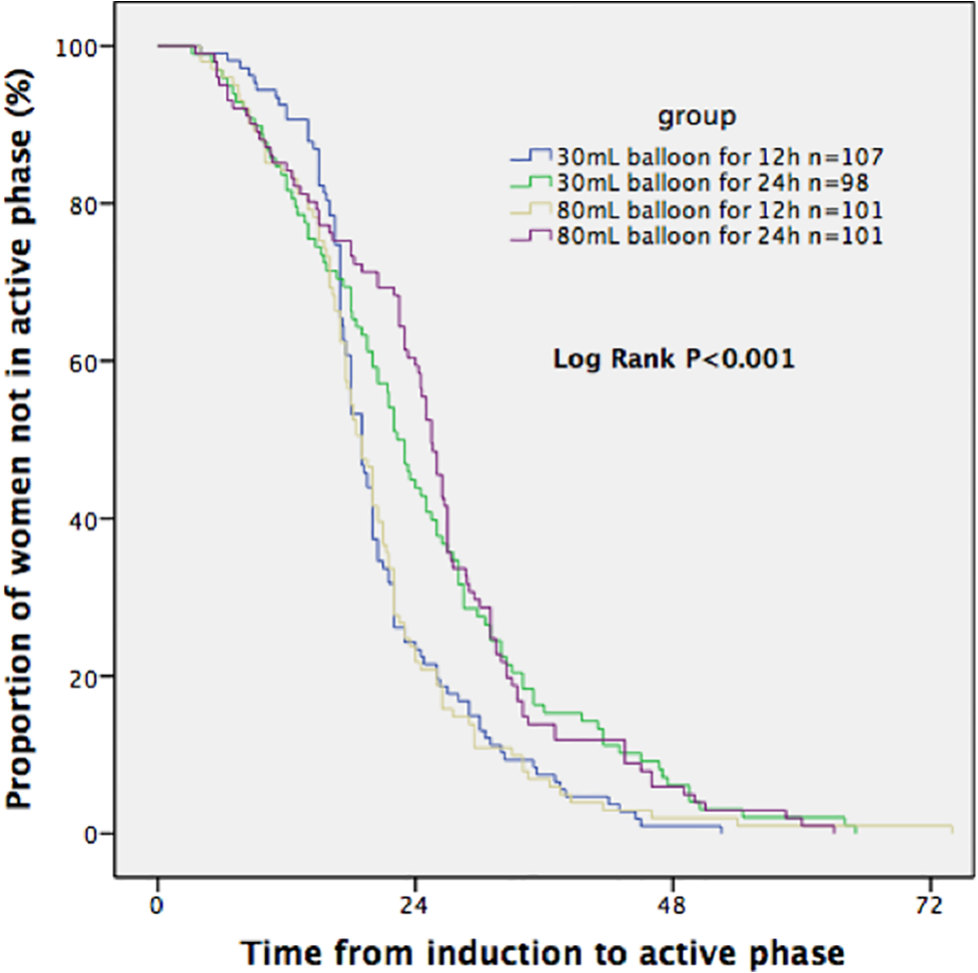
Time from start of induction to the active phase of labor (excluding cesarean deliveries).

**Fig 3:**
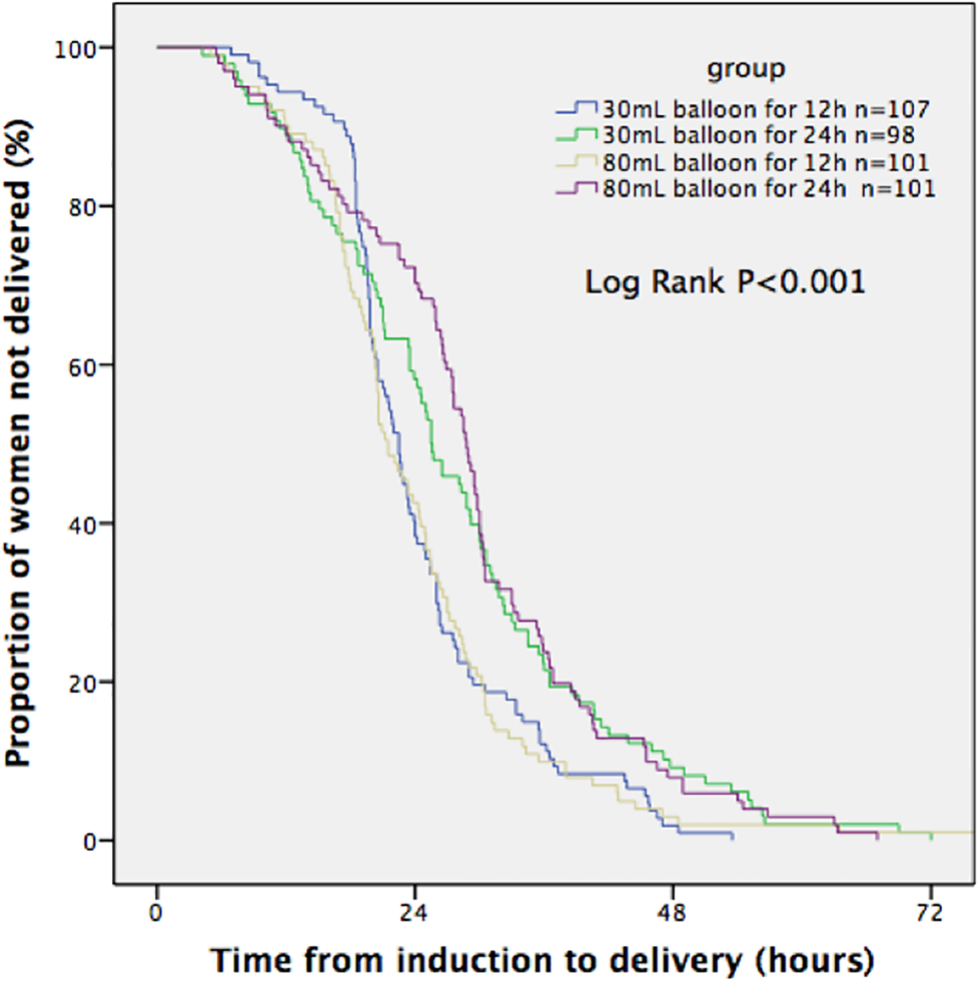
Time from start of induction to vaginal delivery (excluding cesarean deliveries).

Kaplan-Meier survival curves illustrated the fraction of women who progressed to the active phase and gave birth vaginally at a given time after the initiation of cervical ripening in each group (Figs 2 and 3). It demonstrated that the median time from initiation of ripening to active phase and to vaginal delivery was significantly shorter in the two 12-hour Foley catheter groups than in the other two 24-hour Foley catheter groups (Log-rank test, p<0.001). However, on COX multiple regression, variables independently associated with the induction to delivery interval were parity and gestational age.

The frequency of oxytocin administration for induction/augmentation of labor was similar among the groups (Table 2). The maximal effective dose of oxytocin to achieve adequate contraction ranges from 3 to 25 mIU/min, with median dose 15 mIU/min.

Cesarean section rate was lowest (11.6%) in the group with 30-mL balloon for 12 hours although the difference was insignificant among the 4 groups (30-mL/24h: 21.0%, 80-mL/12h: 19.0%, 80-mL/24h: 19.0%, Table 2). The indications for cesarean section were similar among the groups with failure to progress and failed induction being the most common ones. Nine women (1.8%) had cesarean sections for chorioamnionitis and 3 women (0.6%) were operated for fetal distress. There was no difference in assisted vaginal delivery rates among the groups.

There was one case of hyperstimulation in 30-mL balloon group, which occurred before removal of Foley catheter. The incidence of PPH had no statistical difference among the 4 groups (Table 3).

**Table 3:** Maternal and neonatal outcomes.

	30-mL/12h (n = 121)	30-mL/24h (n = 124)	80-mL/12h (n = 125)	80-mL/24h (n = 125)	p
Hyperstimulation n (%)	1 (0.8)	0 (0.0)	0 (0.0)	0 (0.0)	0.244
Chorioamnionitis n (%)	5 (4.1)	3 (2.4)	4 (3.2)	7 (5.6)	0.595
PPH n (%)	29 (24.0)	24 (19.4)	23 (18.4)	29 (23.2)	0.639
Postpartum transfusion n (%)	2 (1.7)	1 (0.8)	2 (1.6)	2 (1.6)	0.932
Birth weight (g, mean ± SD)	3447 ± 393	3458 ± 422	3506 ± 423	3518 ± 402	0.440
Macrosomia > 4000g n (%)	10 (8.3)	8 (6.5)	17 (13.6)	16 (12.8)	0.183
5min Apgar score < 7 n (%)	0 (0.0)	0 (0.0)	1 (0.8)	0 (0.0)	0.619
Neonatal admission n (%)	7 (5.8)	6 (4.8)	2 (1.6)	2 (1.6)	0.152
Ward	6 (5.0)	6 (4.8)	1 (0.8)	2 (1.6)	0.117
NICU	1 (0.8)	0 (0.0)	1 (0.8)	0 (0.0)	0.619
Length of admission (d) (median [range])	3 (3–10)	3 (3–5)	5 (3–7)	3.5 (3–4)	0.757

The incidence of chorioaminonitis was 3.8% overall, and it was similar among the groups (30-mL/12h: 4.1%, 30-mL/24h: 2.4%, 80-mL/12h: 3.2%, 80-mL/24h: 5.6%, p = 0.595, Table 3). None of the women had chorioamnionitis with balloon in situ. Incidence of chorioamnionitis was associated with the time interval from amniotomy to delivery. Within 12 hours after amniotomy, 41.8% of women delivered and only 0.5% of women had choriamnionitis, while rates of chorioamnionitis increased to 5.6% and 7.0% respectively when women delivered between 12 to 24 hours and 24 hours after amniotomy.

Neonatal outcomes, which included Apgar score and neonatal admission, were similar among the groups (Table 3). Three percent of neonates required admission to either neonatal ward or neonatal intensive care unit with no perinatal death during the study. The most common reason for neonatal admission was suspected neonatal infection and the length of admission did not differ significantly among the groups.

Intention-to-treat analysis showed similar results for cesarean section rate and 24-hour vaginal delivery rate. All difference in secondary outcomes among the groups was in agreement with the per-protocol analysis (S1 File).

**Fig 4:**
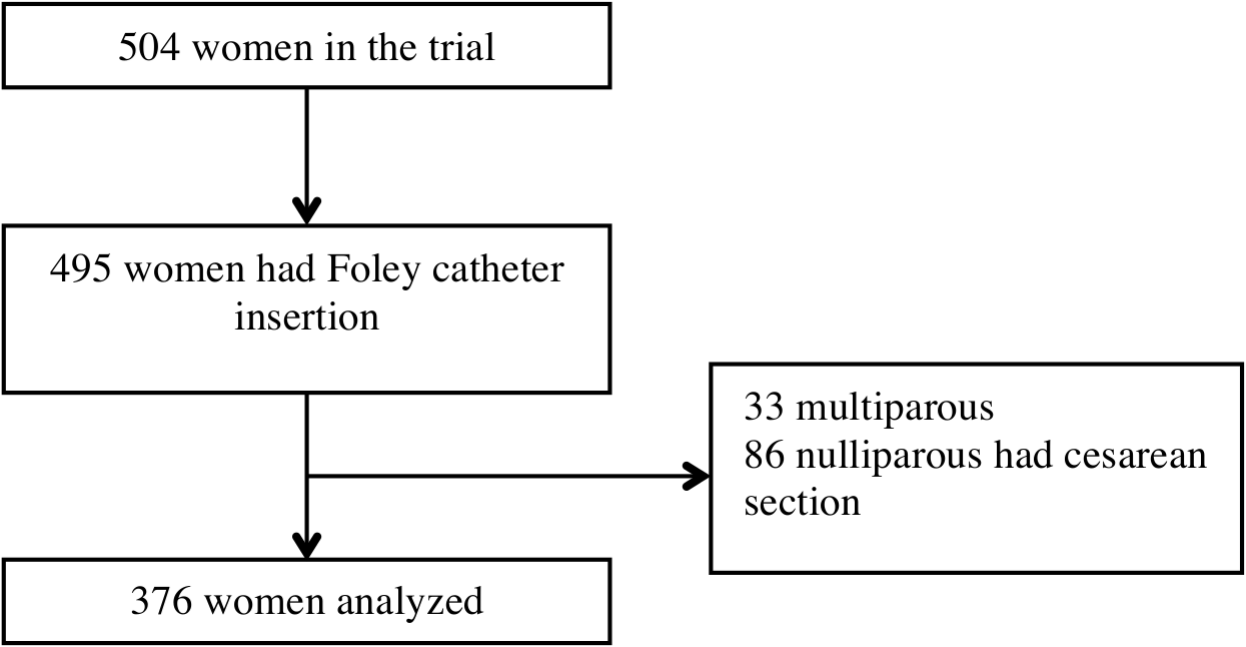
Flowchart of participants included in the analysis of labor patterns.

Three hundred seventy six nulliparous women who had Foley catheter insertion and vaginal deliveries were analyzed to investigate labor patterns (Fig 4). Women with spontaneous labor were included in the control group. Baseline characteristics in the two groups were shown in S2 File. Maternal age and epidural use were similar between the two groups. In the Foley catheter group, gestational age was more advanced (median, 40.3 vs. 40.0 weeks) and pre-delivery body mass index (BMI) was higher (median, 26.8 vs. 26.2 kg/m^2^).

Nulliparous women induced with Foley catheter required a little longer time to progress to 4 cm compared with the spontaneous labor group (median 4.3 hours vs 4.0 hours, p = 0.010, Table 4). There was no difference in the duration of active phase or the second stage of labor; nor was there a difference in the incidence of instrumental vaginal delivery between two groups (Table 4). Women with Foley catheter induction were more likely to have PPH than women with spontaneous onset of labor (19.4% vs 7.7%, p < 0.001). There were no differences in neonatal birth weight or neonatal admission.

**Table 4:** Duration of labor: comparation of spontaneous labor and induced labor.

	Induction with Foley catheter (n = 376)	Spontaneous labor (n = 376)	p
Latent phase (h) (median (95th))	4.3 (10.0)	4.0 (8.6)	0.010
Active phase (h) (median (95th))	2.1 (6.6)	2.0 (6.0)	0.987
First stage (h) (median (95th))	6.8 (14.5)	6.5 (13.0)	0.103
Second stage (h) (median (95th))	0.8 (2.2)	0.8 (1.9)	0.538
Vaginal instrumental delivery n (%)	11 (2.9)	13 (3.5)	0.678
PPH n (%)	73 (19.4)	29 (7.7)	< 0.001
Neonatal weight (g) (mean ± SD)	3452.3 ± 396.8	3456.5 ± 400.1	0.791
Neonatal admission n (%)	8 (2.0)	7 (1.9)	0.794

## Discussion

In this study, we have explored the effect of two balloon sizes and two ripening time limits on the outcomes of term labor induction with Foley catheter balloon. We found that vaginal delivery rate within 24 hours was highest in the group with 30-mL balloon for 12 hours and women induced with Foley catheter had similar duration of active phase and second stage of labor compared with women in spontaneous labor. The overall cesarean section rate was 17.8% and the incidence of chorioamniontis was 3.8%, which suggested that Foley catheter balloon could be safely used in labor induction.

The purpose of induction is to achieve vaginal delivery within a short time, and shorter induction to delivery interval is associated with lower costs and reduced risks of chorioamionitis [14]. Our findings suggest that setting the ripening time limit to 12 hours might increase the proportion of women delivered within 24 hours and generate comparable cesarean section rate. Similar to our study, Cromi et al [9] reported that cutting the maximum time for cervical ripening from 24 to 12 hours would significantly shorten the induction to delivery interval and yield efficacy similar to that of prostaglandin E2 vaginal insert (delivery within 24 hours induction: 24-hour Foley catheter, 21.0%; 12-hour Foley catheter, 59.8%; vaginal prostaglandin E2, 48.5%). We believe that Foley catheter left in place for up to 12 hours brings about cervical changes sufficient for term labor induction, and shorter ripening time is associated with earlier artificial rupture of the membranes and start of oxytocin augmentation, which might be related to quicker labor onset. After adjustment for confounding factors, the effect on vaginal delivery rate within 24 hours induction was insignificant (12-hour vs. 24-hour Foley catheter, OR 2.445, 95%CI 0.733–8.160). However, considering the odds ratio showed a tendency towards higher vaginal delivery rates within 24 hours with 12-hour Foley catheter, we believe that it is worthwhile to do further research with larger sample size to test the hypothesis.

In the present study, we found that ripening of the unfavorable cervix with an 80-mL balloon compared with a 30-mL balloon was not associated with higher rate of vaginal delivery within 24 hours of induction. The cesarean section rate, oxytocin use and neonatal outcomes were comparable among the groups. Our results were different from the report of Levy et al [6], who found that an 80-mL balloon was significantly associated with a higher rate of post-ripening dilation of 3 cm or more in primiparous women, but did not reduce cesarean section rate [6]. The difference in characteristics of participants might be related to the difference between Levy’s trial and our study, and we were concerned that a larger balloon would increase the risk of dislodging the fetal head in some cases although it might be associated with better dilation of cervix. According to our results, longer ripening time and larger balloon size had no benefits on induction outcomes. Thus we favor the use of a 30-mL Foley catheter balloon left in place for a maximum of 12 hours in term labor cervical ripening.

The insertion of a balloon catheter in the cervix might increase the risk of intrapartum infection. In our cohort, around 40 percent of women delivered within 12 hours after amniotomy, whose risks of chorioamnionitis were 0.5%. Over 30 percent of women delivered 12–24 hours after amniotomy, and the incidence of chorioamnionitis was 5.6%, which was relatively low compared with the result of a meta-analysis [15]. Our data showed increased incidence of chorioamnionitis 12 hours after amniotomy, which suggests that shorter induction to delivery interval also provides the benefit of lower risks of intrapartum infection. Balancing the opportunity for vaginal delivery and risks of chorioamnionitis, we considered 24 hours oxytocin induction after rupture of membrane might be allowed for patient with Foley catheter ripening.

The PPH rates were relatively high, from 18.4–24.0% in our study. However, most PPH cases were not severe, since only 1.4% of the women required blood transfusion, which was comparable with the report of Jozwiak et al [4]. The relative higher rate of PPH might be related to the fact that nearly a third of women had risk factors of PPH such as hypertension disorders in pregnancy and gestational or pregestational diabetes and over 80 percent of women had oxytocin for labor induction or augmentation.

Foley catheter insertion may separate the process of ripening of the cervix and start of labor, and is associated with less uterine hyperstimulation with FHR changes when compared with any prostaglandins (RR 0.19; 95% CI 0.08–0.43; 9 studies, 1931 women) [1]. In our trial, we recorded one case of uterine hyperstimulation in 30-mL balloon group with fetal heart rate deceleration when balloon catheter was in situ. Although hyperstimulation was relatively uncommon, meticulous monitoring of FHR and contractions was recommended when balloon catheter was used.

Another finding of our study is that women who achieved vaginal delivery after Foley catheter induction have similar labor patterns compared with women in spontaneous labor. Nulliparous women induced with Foley catheter required a little longer time to progress to 4 cm compared with women in the spontaneous labor group; however, after 4 cm, both groups had similar rates of cervical dilation. We believe that Foley catheter, which dilates the cervix mechanically, is associated with a delayed transition to active labor. The clinical significance of this observation is that diagnosis of failed progress of labor should not be made until the active phase of labor in women induced with the Foley catheter.

The current study had some limitations. First, the study lacked sufficient power to show significant treatment differences in secondary outcomes such as cesarean section rate and maternal complications. Second, it was an open label research and the method of cervical ripening might have affected the obstetricians’ decision. Third, we did not record women’s satisfaction with different balloon volume and ripening time; however, none of the participants withdrew from the trial for discomfort during and after balloon insertion, which indicated the safety of device placement.

In summary, we included women with a variety of indications for induction in the present trial, which suggested that Foley catheter balloon could be safely used in cervical ripening for both obstetrical and medical indications. Furthermore, considering the low cost and easy storage of the Foley catheter, we believe it could be used for low-resource settings, such as rural and county hospitals in China.

## Supporting Information

S1 CONSORT Checklist CONSORT Checklist. (DOC)

S1 File Supplementary data of intention-to-treat analysis results. (DOCX)

S2 File Baseline characteristics of participants included in the analysis of labor patterns. (DOCX)

S3 File Data of the trial participants. (XLSX)

S1 Protocol Trial Protocol in Chinese. (DOCX)

S2 Protocol Trial Protocol in English. (DOCX)
