# Supplementary material for: Foley Catheter for Induction of Labor at Term: An Open-Label, Randomized Controlled Trial
Source: PLoS One. 2015 Aug 31;10(8):e0136856. doi: 10.1371/journal.pone.0136856 (PMC4556187; doi:10.1371/journal.pone.0136856)
Supplement: S1 File — (DOCX) [file pone.0136856.s002.docx]

**Table 1. Baseline characteristics of subjects (intention-to-treat set).**

|  | 30-mL/12h | 30-mL/24h | | 80-mL/12h | | 80-mL/24h | p |
| --- | --- | --- | --- | --- | --- | --- | --- |
|  | (n = 126) | (n = 126) | | (n = 126) | | (n = 126) |  |
| Maternal age (years; mean ± SD) | 28.0 ± 3.4 | 28.3 ± 3.5 | | 28.5 ± 3.4 | | 28.3 ± 3.5 | 0.716 |
| Parity n (%) |  |  | |  | |  | 0.668 |
| 0 | 119 (94.4) | 119 (94.4) | | 114 (90.5) | | 119 (94.4) |  |
| 1 | 7 (5.6) | 6 (4.8) | | 11 (8.7) | | 7 (5.6) |  |
| 2 | 0 (0.0) | 1 (0.8) | | 1(0.8) | | 0 (0.0) |  |
| Bishop score (median [IQR]) | 4 (4-5) | 4 (4-5) | | 4 (4-5) | | 4(4-5) | 0.270 |
| GA (weeks; median [IQR]) | 40.1 (39.3-41.0) | 41.0 (39.4-41.0) | | 40.0 (39.1-41.0) | | 40.4 (39.6-41.0) | 0.400 |
| Indications for induction n (%) | | |  | |  | |  |
| GA ≥41 weeks | 61 (48.4) | 73 (57.9) | | 55 (43.7) | | 56 (44.4) |  |
| GDM/Diabetes | 44 (34.9) | 32 (25.4) | | 32 (25.4) | | 44 (34.9) |  |
| HDP | 8 (6.3) | 7 (5.6) | | 19 (15.1) | | 13 (10.3) |  |
| FGR | 1 (0.8) | 2 (1.6) | | 8 (6.3) | | 3 (2.4) |  |
| Oligohydramnion | 10 (7.9) | 8 (6.3) | | 9 (7.1) | | 9 (7.1) |  |
| Other | 2 (1.6) | 4 (3.2) | | 3 (2.4) | | 1 (0.8) |  |

Abbreviation: GA, gestational age. GDM, gestational diabetes mellitus. HDP, hypertensive disorders in pregnancy. FGR, fetal growth restriction.

**Table 2. Induction to delivery interval and mode of delivery (intention-to-treat set).**

|  | 30-mL/12h | 30-mL/24h | 80-mL/12h | 80-mL/24h | p |
| --- | --- | --- | --- | --- | --- |
|  | (n＝126) | (n＝126) | (n＝126) | (n＝126) |  |
| Vaginal delivery within 24 hours (h) n (%) | 68 (54.0) | 41 (32.5) | 59 (46.8) | 30 (23.8) | <0.001 |
| Time to delivery (h) (median [IQR]) | 23.3 (19.5-33.4) | 29.2 (20.7-42.5) | 23.6 (18.0-30.5) | 29.4 (24.2-37.3) | <0.001 |
| Time to active phase (h) (median [IQR])* | 19.0 (16.5-23.8) | 23.0 (14.8-32.0) | 19.0 (15.4-23.1) | 25.5 (17.6-31.1) | <0.001 |
| Time to vaginal delivery (h) (median [IQR])* | 22.6 (19.2-28.0) | 25.7 (18.6-35.8) | 21.4 (17.3-28.4) | 28.8 (22.1-35.8) | 0.001 |
| Mode of delivery n (%) |  |  |  |  | 0.560 |
| Spontaneous | 109 (86.5) | 97 (77.0) | 99 (78.6) | 99 (78.6) |  |
| Vaginal instrumental | 3 (2.4) | 3 (2.4) | 3 (2.4) | 3 (2.4) |  |
| Cesarean section | 14 (11.1) | 26 (20.6) | 24 (19.0) | 24 (19.0) |  |
| Indications for Cesarean section n (%) | |  |  |  | 0.514 |
| Failed induction | 7 (50.0) | 10 (38.5) | 6 (25.0) | 12 (50.0) |  |
| Failure to progress | 5 (35.7) | 11 (42.3) | 15 (62.5) | 10 (41.6) |  |
| Fetal distress | 1 (7.1) | 2 (7.7) | 0 (0.0) | 0 (0.0) |  |
| Chorioamionitis | 1 (7.1) | 3 (11.5) | 3 (12.5) | 2 (8.3) |  |
| Indications for vaginal instrumental delivery n (%) | | |  |  | 0.721 |
| Failure to progress | 3 (100.0) | 2 (66.7) | 2 (66.7) | 2 (66.7) |  |
| Fetal distress | 0 (0.0) | 1 (33.3) | 1 (33.3) | 1 (33.3) |  |
| Oxytocin use n (%) | 109 (86.5) | 111 (88.1) | 108 (85.7) | 114 (90.5) | 0.672 |
| Epidural analgesia n (%) | 25 (19.8) | 27 (21.4) | 15 (11.9) | 24 (19.0) | 0.208 |

* Excluding cesarean deliveries.

**Table 3. Maternal and neonatal outcomes (intention-to-treat set).**

|  | 30-mL/12h | 30-mL/24h | 80-mL/12h | 80-mL/24h | p |
| --- | --- | --- | --- | --- | --- |
|  | (n＝126) | (n＝126) | (n＝126) | (n＝126) |  |
| Hyperstimulation n (%) | 1 (0.8) | 0 (0.0) | 0 (0.0) | 0 (0.0) | 1.000 |
| Chorioamnionitis n (%) | 5 (4.0) | 3 (2.4) | 4 (3.2) | 7 (5.6) | 0.590 |
| PPH (>500mL) n (%) | 29 (23.0) | 26 (20.6) | 23 (18.3) | 29 (23.0) | 0.759 |
| Postpartum transfusion n (%) | 2 (1.7) | 1 (0.8) | 2 (1.6) | 2 (1.6) | 0.933 |
| Birth weight (g; mean ± SD) | 3438±392 | 3460±422 | 3507±420 | 3519±401 | 0.347 |
| Macrosomia > 4000g n (%) | 10 (7.9) | 9 (7.1) | 17 (13.5) | 16 (12.7) | 0.301 |
| 5 min Apgar score < 7 n (%) | 0 (0.0) | 0 (0.0) | 1 (0.8) | 0 (0.0) | 1.000 |
| Neonatal admission n (%) | 7 (5.6) | 6 (4.8) | 2 (1.6) | 2 (1.6) | 0.176 |
| Ward | 6 (4.8) | 6 (4.8) | 1 (0.8) | 2 (1.6) | 0.127 |
| NICU | 1 (0.8) | 0 (0.0) | 1 (0.8) | 0 (0.0) | 1.000 |
| Length of admission (d; median [range]) | 3 (3-10) | 3 (3-5) | 5 (3-7) | 3.5 (3-4) | 0.757 |
