# Supplementary material for: Foley Catheter for Induction of Labor at Term: An Open-Label, Randomized Controlled Trial
Source: PLoS One. 2015 Aug 31;10(8):e0136856. doi: 10.1371/journal.pone.0136856 (PMC4556187; doi:10.1371/journal.pone.0136856)
Supplement: S2 File — (DOCX) [file pone.0136856.s003.docx]

**Table 1. Baseline characteristic of women included in the analysis of labor patterns**

|  | Induction with Foley catheter (n＝376) | Spontaneous labor (n=376) | p |
| --- | --- | --- | --- |
| Maternal age (years; mean ± SD) | 28.0 ± 3.2 | 27.6 ± 3.0 | 0.197 |
| BMI before delivery (kg/m^2^; median [IQR]) | 26.8 (25.0-29.2) | 26.2 (24.3-28.0) | <0.001 |
| GA (weeks; median [IQR]) | 40.3 (39.3-41.0) | 40.0 (39.3-40.6) | <0.001 |
| Epidural analgesia n (%) | 73/376 (19.4) | 61/376 (16.2) | 0.253 |
| Oxytocin use n (%) | 291/376 (77.4) | 96/376 (25.5) | <0.001 |
| Maximum dose of oxy (mIU/min; median [range]) | 15 (3-25) | 12 (3-25) | 0.292 |
