# Supplementary material for: Foley Catheter for Induction of Labor at Term: An Open-Label, Randomized Controlled Trial
Source: PLoS One. 2015 Aug 31;10(8):e0136856. doi: 10.1371/journal.pone.0136856 (PMC4556187; doi:10.1371/journal.pone.0136856)
Supplement: S1 Protocol — (DOCX) [file pone.0136856.s005.docx]

**Foley尿管水囊用于足月妊娠引产的多中心随机对照临床研究（研究方案）**

**伦理委员会申报材料**

**南京市鼓楼医院**

**2013年1月**

**主要研究者 胡娅莉教授**

1. **研究意义和背景**

妊娠晚期引产是产科处理高危妊娠最常用的手段之一。引产是否成功主要取决于宫颈成熟度。常用的药物促宫颈成熟方法包括口服或阴道内使用前列腺素药物。水囊可以通过机械刺激宫颈管达到促宫颈成熟的目的。Foley尿管水囊价格低廉，易获得，易储存，子宫过度刺激发生率低，引产的成功率与药物引产近似[1]。

不同的研究中水囊大小和放置时间均有差异。文献中不同大小的水囊（30-80mL）对于引产至分娩时间间隔以及剖宫产率的影响存在不一致的报道[2-4]。有的研究者设定水囊促熟的最大时限，有的研究者等待水囊自行脱落［4-5］。Cromi[6]对不同的水囊放置时限进行了研究，结果发现将水囊促熟时限由24小时降低至12小时，可以明显提高24小时内阴道分娩率。在我们既往的研究中，受试者均为初产妇，水囊的容量为80mL，放置时间为24小时，24小时内分娩率相对较低[7]。在今后的研究中，我们希望提高水囊引产的效率，也就是缩短引产至分娩时间。因此我们希望通过随机对照临床研究探索足月妊娠促宫颈成熟合适的水囊大小及放置时间。

1. **研究目的**

研究目的：探索足月妊娠促宫颈成熟合适的水囊大小及放置时间。具体研究目的包括以下几点：

- 1. 两种不同大小的水囊和两种促熟时限对水囊引产结局的影响。
  2. 水囊引产的母儿并发症。
  3. 水囊引产和自然临产产程的比较。

1. **研究设计**

1、前瞻性、开放、多中心、随机平行对照临床研究。满足入组／排除标准的妇女根据水囊大小和促熟时限的不同按照1:1:1:1的比例分为以下4组（见表 1）。本研究对研究者和受试者均不设盲。

表1. 分组情况

| 分组 | 干预 | 样本量 |
| --- | --- | --- |
| 1 | 30mL水囊×12小时 | 126 |
| 2 | 30mL水囊×24小时 | 126 |
| 3 | 80mL水囊×12小时 | 126 |
| 4 | 80mL水囊×24小时 | 126 |

2、随机分组：本研究采用区组随机化方法，利用SAS软件产生随机数字表。由于本地区引产的妇女中初产妇约占95%，故未对产次进行分层。随机数字表由统计师许碧云提供并保存。在研究开始前，由未参与研究的两名医生将随机序列号装入不透明的信封中，由专人保管。临床研究者在试验过程中不能接触随机数字表。

3、入选标准：妊娠37周以上，单胎、头位、胎膜未破、宫颈Bishop评分<6分、有引产指征。排除标准：母亲年龄<18岁，双胎或多胎妊娠、骨盆异常、胎位异常、前置胎盘、生殖道炎症、死胎、GBS（+）。

4、主要观察指标：24小时内阴道分娩率。

5、次要观察指标：剖宫产率、阴道助产率，剖宫产指征，阴道助产指征，引产至分娩时间间隔，引产至活跃期时间间隔，分娩镇痛率，缩宫素使用率，缩宫素剂量、子宫过度刺激、母亲绒毛膜羊膜炎，产后出血（产后24小时内失血≥500mL），产后输血，宫口扩张和先露下降，新生儿体重，Apgar评分小于7分，新生儿住院率及住院指征。

6、研究时间：2013年2月至2014年6月

7、预计例数：我们既往对初产妇的研究中使用80mL水囊促宫颈成熟，放置时限24小时，24小时分娩率28.1%。文献中数据提示水囊引产24小时内阴道分娩率为48-66%。我们希望通过此项研究寻找到水囊大小与放置时间的最优组合，将24小时内分娩率提高20%，也就是48%。根据多个样本率比较的样本含量计算公式 (α error=5%, power=80%)：

$$n=2\lambda/{(2\sin^{-1} \sqrt{p_{max}}-2\sin^{-1} \sqrt{p_{min}})}^{2}$$

计算每组需126例。

8. 产程研究 为了比较Foley尿管引产妇女的产程和自然临产妇女的产程（初产妇），我们将在南京市鼓楼医院前瞻性地采集自然临产妇女的临床资料（样本量与水囊引产组1:1）。自然临产组妇女的入组标准为：初产妇、单胎头位、入院时宫口开大<2cm，自然临产。排除标准为：早产、胎儿畸形以及剖宫产分娩。受试者需要签订书面的知情同意后医生方可采集其临床资料。自然临产组与水囊引产组年龄上需配对，孕周上相差在7天之内。

1. **研究单位**

南京鼓楼医院是一所三级甲等综合性医院，年分娩量4000余例，预计完成296例。无锡市妇幼保健院是一所三级甲等妇幼专科医院，年分娩量5000余例，预计完成52例。泰兴市人民医院是一所三级乙等综合性医院，年分娩量2000余例，预计完成52例。昆山市第一人民医院是一所三级乙等综合性医院，年分娩量2000余例，预计完成52例。金坛市人民医院是一所二级甲等综合性医院年分娩量1300例，预计完成52例（见表2）。

表2. 研究单位

| 研究单位 | 预计完成的例数 |
| --- | --- |
| 南京鼓楼医院（组长） | 296 |
| 无锡市妇幼保健院 | 52 |
| 泰兴市人民医院 | 52 |
| 昆山市第一人民医院 | 52 |
| 金坛市人民医院 | 52 |

1. **经费来源**

本研究的经费来自国家临床重点专科建设项目(2011271)。

1. **研究实施**

**1、水囊引产实施方案**

**入**组流**程**

拟行引产的足月妊娠妇女入院后，首先由产科医生评估引产的指征、禁忌症。产科医生将核对预产期、估算胎儿体重、进行阴道检查评估骨盆大小及宫颈Bishop评分[8]。行白带检查排除阴道炎。回顾孕期超声检查结果，排除前置胎盘。经过筛选后，满足入组/排除标准的妇女由产科医师告知本研究的目的、流程及风险。愿意参加临床试验的产妇签订知情同意书（一式两份）接受Foley尿管水囊引产。不愿参加临床研究的妇女按照各单位引产常规处理。

随机化

获得产妇知情同意后，产科医生和另一名研究者共同打开装有随机序列号的信封，获得分组信息。入组时产科医师将记录病史资料和引产指征，核实孕周，记录Bishop评分并填写引产安全核查表（附件1）。

水囊放置和取出

水囊放置: 放置前孕妇排空膀胱，取膀胱截石位，0.5%碘伏消毒外阴及阴道。无菌窥器置入阴道内暴露宫颈，借助卵圆钳将16F Foley尿管置入宫颈，使尿管球囊通过宫颈内口，向水囊内注射无菌生理盐水，轻轻回拉水囊，确定其在宫颈内口上方后夹闭导管末端，拉直并胶带固定于大腿内侧。放置结束后行胎心监测。第1组及第2组的妇女置入30mL水囊，最大促熟时限分别为12小时和24小时。第3组及第4组妇女置入80mL水囊，最大促熟时限分别为12小时和24小时。

出现以下情况需考虑退出研究：（1）水囊放置失败；（2）放置时或放置后产妇阴道流血或严重不适；（2）受试者或主治医师要求退出研究。对于退出研究者的妇女继续记录母儿结局，纳入意向性分析集。

取出水囊指征包括：(1)达到促熟时限，(2) 胎膜破裂，(3)水囊自行排出，(4)临产，(5)子宫过度刺激或胎儿宫内窘迫。

促宫颈成熟后的引产流程

促宫颈成熟后的引产流程：水囊排出或取出后未临产者，立即人工破膜，破膜后30min无规律宫缩则给予小剂量缩宫素(1mIU/min起，每20 min增加2mIU/min直到宫缩达到200-250蒙氏单位，最大缩宫素剂量25mIU/min)静脉滴注引产。如果破膜失败，给予小剂量缩宫素(1mIU/min起，最大剂量25mIU/min)静脉滴注引产，6小时后再次尝试破膜。一旦进入活跃期，则停用缩宫素，每6小时一次记录母体的心率、血压和体温，行持续电子胎心监护。

产程管理

受试者临产后按各中心的产程管理常规进行处理。产程中每6小时记录产妇体温、心率、血压。产妇进入活跃期（宫口4cm）后需行持续电子胎心监护。如果产妇因产程异常、胎心异常或其他母儿因素拟行剖宫产分娩，需由主治或以上级别医师作出临床决策。

产后管理

受试者分娩后按各中心产后管理常规进行处理。受试者出院48小时内由产科医生填写《水囊引产研究病例表格》（附件 2）。受试者产后30天，产科医生电话随访产后母儿并发症（产褥感染、晚期产后出血及新生儿的情况）。

不良反应

子宫过度刺激定义为10min内宫缩≥6次或一次宫缩持续时间>2min伴或不伴胎心率异常[9]。

引产失败定义为破膜且缩宫素引产24小时后仍未进入活跃期。活跃期宫口不再扩张达4小时以上诊断为活跃期停滞。

产程异常定义为活跃期规律宫缩4小时宫口扩张无进展。

绒毛膜羊膜炎定义为母亲体温≥38°C伴以下情况之一者：母亲心率>100次／分，胎儿心动过速，子宫压痛，羊水异味[10]。

产后出血定义为胎儿娩出后24小时内失血量超过500ml。失血量的测量使用称重法（失血量＝（接血敷料湿重（g）－敷料干重（g））／1.05g/ml）和容积法（失血量＝接血盆或吸引器中的血液容积）。

**2、自然临产组的数据采集**

**入**组流**程**

当水囊引产组出现一例初产妇经阴道分娩后，研究者选取一例年龄配对，分娩日期最接近的自然临产产妇入组。自然临产组的妇女与水囊引产组相比孕周上相差在7天之内。入组标准为：初产妇、单胎头位、入院时宫口开大<2cm，自然临产，阴道分娩。排除标准为：早产、胎儿畸形以及剖宫产分娩。自然临产组产程中可能会接受人工破膜或缩宫素增缩。满足入组／排除标准者由产科医生告知研究的目的和方法。受试者需要签订书面的知情同意后医生方可采集其临床资料（仅在鼓楼医院收集）。

**数据采集**

受试者出院48小时内由产科医生填写《自然临产研究病例表格》（附件 3）。受试者产后30天，产科医生电话随访产后母儿并发症（产褥感染、晚期产后出血及新生儿的情况）。

**3、年度计划**

1. **准备阶段（2012年6月至2013年1月）**

在准备阶段，研究者制定研究计划，对参与研究的各中心引产和分娩的情况进行调查。培训研究者宫颈评分及水囊放置的操作流程。规范各个中心缩宫素引产的操作流程。各中心进行预试验熟悉水囊引产的流程。确定研究管理方案和管理人员。

1. **实施阶段（2013年2月至2014年6月）**

本阶段进行随机对照临床研究。受试者入组并随访至产后1月。一旦完成课题所需的样本量即停止入组。

1. **数据分析（2014年7月至2014年12月）**

本阶段完成数据分析，总结报告，撰写论文。

**七、统计分析**

本研究采用SPSS 17.0软件分别对意向性分析集和符合方案集进行统计分析。正态分布的数据使用平均数和标准差；偏态分布的数据采用中位数、四分位距和全距。分类指标的描述用各类的例数及百分数。计量资料的四组比较采用方差分析或者秩和检验。计数资料的四组比较采用卡方检验或Fisher精确概率。引产至分娩时间间隔用Kaplan-Meier生存曲线表示，进行Logrank检验。P<0.05提示有显著差异。

**八、研究参加人员**

|  | 姓 名 | 单 位 | 承担工作 |
| --- | --- | --- | --- |
| 主要负责人 | 胡娅莉 | 南京鼓楼医院 | 主要研究者 |
|  | 胡玲卿 | 无锡市妇幼保健院 | 分中心负责人 |
|  | 徐彪 | 泰兴市人民医院 | 分中心负责人 |
|  | 刘琴 | 昆山市人民医院 | 分中心负责人 |
|  | 丁屹 | 金坛市人民医院 | 分中心负责人 |
| 参  加  项  目  研  究  人  员 | 王志群 | 南京鼓楼医院 | 分中心负责人 |
|  | 戴毅敏 | 南京鼓楼医院 | 数据采集 |
|  | 顾 宁 | 南京鼓楼医院 | 数据采集 |
|  | 许碧云 | 南京鼓楼医院 | 数据统计 |
|  | 郑明明 | 南京鼓楼医院 | 数据采集 |
|  | 凌静娴 | 南京鼓楼医院 | 数据采集 |
|  | 张 蕾 | 南京鼓楼医院 | 数据采集 |
|  | 仇黎丽 | 南京鼓楼医院 | 监察员 |
|  | 翁 侨 | 南京鼓楼医院 | 数据管理 |

**九、不良事件的处理**

1、宫缩过频的处理：1）取出水囊；2）嘱孕妇左侧卧位；3）持续胎心监护；4）必要时给予宫缩抑制剂；5）宫缩过频伴胎心监护III类图形应考虑剖宫产。

2、胎心监护异常的处理：1）停用任何可以诱发宫缩的药物或其他因素；2）阴道检查了解有无脐带脱垂、宫口扩张过速或胎头下降过快；3）改变体位至左侧卧或右侧卧；4）监测母亲血压以排除低血压（扩容/肾上腺素）；5）评估是否存在宫缩过频；6）吸氧；7）必要时使用宫缩抑制剂；8）确定胎儿窘迫估计短时间内不能经阴道结束分娩者剖宫产。

3、绒毛膜羊膜炎的处理：体温≥38时应使用广谱抗生素：头孢西丁2g，6小时一次；头孢西丁过敏者使用克林霉素0.9g，8小时一次。

**十、 研究病历**

受试者入组后，产科医师负责填写研究病例表格。研究病例表格中不包含受试者的个人信息（姓名、地址及电话）。各中心的研究病例表格汇总至南京鼓楼医院，由鼓楼医院的数据管理者录入数据，如需修改数据须与记录临床资料的研究者核实并汇报监察员。研究结束后，由统计人员完成对数据的统计分析。

**参考文献**

1. Jozwiak M, Bloemenkamp KW, Kelly AJ, et al. Mechanical methods for induction of labour Cochrane Database Syst Rev. 2012, 14; 3:CD001233.
2. Pennell CE, Henderson JJ, O’Neill MJ, et al. Induction of labour in nulliparous women with an unfavourable cervix: a randomised controlled trial comparing double and single balloon catheters and PGE2 gel. BJOG, 2009; 116: 1443–52.
3. Levy R, Kanengiser B, Furman B, et al. A randomized trial comparing a 30-mL and an 80-mL Foley catheter balloon for preinduction cervical ripening. Obstet Gynecol 2004; 191(5):1632–6.
4. Delaney S, Shaffer B, Cheng Y, et al. Labor Induction With a Foley Balloon Inflated to 30 mL Compared With 60 mL. Obstet Gynecol 2010; 115:1239-1245.
5. [Prager M](http://www.ncbi.nlm.nih.gov/pubmed?term=Prager%20M%5BAuthor%5D&cauthor=true&cauthor_uid=18715244), [Eneroth-Grimfors E](http://www.ncbi.nlm.nih.gov/pubmed?term=Eneroth-Grimfors%20E%5BAuthor%5D&cauthor=true&cauthor_uid=18715244), [Edlund M](http://www.ncbi.nlm.nih.gov/pubmed?term=Edlund%20M%5BAuthor%5D&cauthor=true&cauthor_uid=18715244), [Marions L](http://www.ncbi.nlm.nih.gov/pubmed?term=Marions%20L%5BAuthor%5D&cauthor=true&cauthor_uid=18715244). A randomised controlled trial of intravaginal dinoprostone, intravaginal misoprostol and transcervical balloon catheter for labour induction. BJOG. 2008;115(11):1443-50.
6. Cromi A, Ghezzi F, Agosti M, et al. Is transcervical Foley catheter actually slower than prostaglandins in ripening the cervix? A randomized study. Am J Obstet Gynecol 2011; 204:338.e1-7.
7. Zheng MM, Hu YL, Zhang SM, Ling JX, Wang ZQ. Trans-cervical Foley catheter balloon versus vaginal prostaglandin E2 suppository for cervical ripening and induction of labor: a prospective randomized controlled trial. Chinese Journal of Perinatal Medicine. 2011; 14: 648-652.
8. Bishop EH. Pelvic scoring for elective induction. Obstet Gynecol 1964; 24:266–8.
9. ACOG Committee. [ACOG Practice Bulletin No. 107: Induction of labor.](http://www.ncbi.nlm.nih.gov/pubmed/19623003) Obstet Gynecol. 2009; 114:386-97.
10. [Hauth JC](http://www.ncbi.nlm.nih.gov/pubmed?term=Hauth%20JC%5BAuthor%5D&cauthor=true&cauthor_uid=4011072), Gilstrap LC 3^rd^, Hankins GD, et al. Term maternal and neonatal complications of acute chorioamnionitis.Obstet Gynecol. 1985;66(1):59-6

### 附件一 引产安全核查表

| 日期 | 姓名 | 年龄 |
| --- | --- | --- |
| 住院号 | 孕／产次 | EDC |
| 孕周 | 引产指征 |  |

- 经过核实，孕龄≥39周
  - 20周之内的超声检查证实孕龄≥39周
  - 距离第一次多普勒胎心记录≥30周
- 如果为39周前的引产，有医学指征且获得上级医生同意
- 单胎头先露
- 胎膜未破
- 骨盆正常
- 胎盘位置正常
- 白带正常
- 胎心监护NST有反应
- 估计胎儿体重
- GBS
- 已了解孕妇病史及检查结果
  - 药物过敏史
  - 内科合并症
  - 产科合并症
  - 有辅助检查结果（血常规、血型、心电图等）
- 产妇知情同意
- Bishop评分
- 引产方式

| - - 30ml水囊×12h | - - 80ml水囊×12h |
| --- | --- |
| - - 30ml水囊×24h | - - 80ml水囊×24h |

医生签名

**附件二 水囊引产研究病例表格（CRF表）**

| 出生年月 | 年 月 | | | 住院号 | | | |  |
| --- | --- | --- | --- | --- | --- | --- | --- | --- |
| 身高 | cm | | | 目前体重 | | | | Kg |
| 目前孕周 | 周 天 | | | 产次 | | | |  |
| 引产指征 | - □延期妊娠（≥41周） - □胎儿宫内生长受限 - □妊娠合并内科疾病 | | | - □GDM／DM   □羊水过少 | | | | - □妊娠期高血压疾病   □ICP  □其他 |
| 引产方式 | □ 30ml水囊×12h  □ 80ml水囊×12h | | | □30ml水囊×24h  □80ml水囊×24h | | | |  |
| 引产日期 | 月 日 时 分 | | | 引产前Bishop | | | |  |
| 水囊取出日期 | 月 日 时 分 | | | 引产后Bishop | | | |  |
| 水囊取出原因 | □自行脱落 | | | □达到时限 | | | | □胎心监护异常 |
|  | □宫缩过频、过强 | | | □胎膜破裂 | | | | □阴道流血超过月经量 |
|  | □临产 | | | □其他不良反应 | | | |  |
| 取水囊后引产方式 | □人工破膜 | | | 日期 | | | | 月 日 时 分 |
|  | - 缩宫素 - 缩宫素最大剂量 | | | 日期 | | | | 月 日 时 分 |
|  | □其他 | | | 日期 | | | | 月 日 时 分 |
| 产程资料 |  | | |  | | | |  |
| 时间 | | | 宫口 | | | 先露 | | |
|  | | |  | | |  | | |
|  | | |  | | |  | | |
|  | | |  | | |  | | |
|  | | |  | | |  | | |
|  | | |  | | |  | | |
|  | | |  | | |  | | |
|  | | |  | | |  | | |
|  | | |  | | |  | | |
|  | | |  | | |  | | |
|  | | |  | | |  | | |
|  | | |  | | |  | | |
|  | | |  | | |  | | |
| 分娩时间 | | 月 日 时 分 | | |  | |  | |
| 分娩方式 | | □阴道分娩 | | | □剖宫产 | | □产钳助产 | |
| 剖宫产手术指征 | | □引产失败 | | | □产程异常 | | □其他 | |
|  | | □胎儿宫内窘迫（□胎心异常 □羊水粪染） | | | | | | |
| 产钳助产指征 | |  | | |  | |  | |
| 产时镇痛 | | □硬膜外镇痛 | | |  | |  | |
| 产后出血 | | mL | | | 产后输血 | | □ 是 □否 | |
| 母亲感染 | | □无 | | | □绒毛膜羊膜炎 | | □产后子宫内膜炎 | |
| 子宫过度刺激 | | □无 □有 | | | | |  | |
|  | | □ 子宫破裂 | | | □ 胎盘早剥 | |  | |
| 其他产时并发症  产后诊断 | |  | | | 产妇住院天数 | | 天 | |
| 新生儿体重 | |  | | | 新生儿评分 | | □1min 5min | |
| 新生儿住院 | | □特婴室 □NICU | | | □无 | |  | |
| 住院指征 | | □新生儿感染 | | | □新生儿窒息 | | □胎粪吸入性肺炎 | |
|  | | □新生儿呼吸窘迫综合征 | | | □低出生体重 | | □缺血缺氧脑病 | |
|  | | □其他 | | | □新生儿死亡 | |  | |

新生儿住院时间 天

**附件三 自然临产的研究病例表格**

**母亲资料**（足月单胎头位初产妇、自然临产、阴道分娩、入院宫口< 2cm；排除引产、多胎、死胎、中孕引产史、胎位不正、新生儿畸形、剖宫产分娩史）

| 住院号 |  | 初产妇 | □是 |  |  |
| --- | --- | --- | --- | --- | --- |
| 年龄（y） |  | 身高（cm） |  | 体重（kg） |  |
| 入院时间 |  | 合并症 |  |  |  |
| 入院宫口 |  | 颈管（cm） |  | 先露 |  |
| oxy增缩 | □是□否 | 时间（h） |  | Oxy最大量 |  |
| 硬膜外 | □是 |  |  |  |  |
| 分娩孕周 |  | 新生儿体重 |  | 产后出血 |  |
| 新生儿住院 |  |  |  | 产后输血 |  |
| 助产 | □是□否 | 助产指征 | □产程 | □胎窘 | □其他 |

**产程资料**

| 时间 | 宫口 | 先露 |
| --- | --- | --- |
|  |  |  |
|  |  |  |
|  |  |  |
|  |  |  |
|  |  |  |
|  |  |  |
|  |  |  |
|  |  |  |
|  |  |  |
|  |  |  |
|  |  |  |
