# Supplementary material for: Foley Catheter for Induction of Labor at Term: An Open-Label, Randomized Controlled Trial
Source: PLoS One. 2015 Aug 31;10(8):e0136856. doi: 10.1371/journal.pone.0136856 (PMC4556187; doi:10.1371/journal.pone.0136856)
Supplement: S2 Protocol — (DOCX) [file pone.0136856.s006.docx]

**Foley Catheter for Induction of Labor at Term:**

**a Multi-Center Randomized Controlled Trial**

**(Study Protocol)**

**Protocol Submission To**

**Nanjing Drum Tower Hospital**

**Institutional Review Board**

**January 2013**

**Principal Investigator**

**Prof. Yali Hu**

**1. Background**

Labor induction is a frequently used method in the management of high-risk pregnancy. Both medical and mechanical methods have been applied to induction of labor. Foley catheter insertion has been proved as effective as prostaglandins in achieving delivery within 24 hours without increasing the risk of cesarean section [1]. In the mean time, Foley catheter use provides the advantage of low risk of hyperstimulation and low cost of the devices.

There was considerable heterogeneity in mechanical ripening protocols in literature. Balloon inflation sizes of 30-80mL have been compared with inconsistent results regarding induction-to-delivery time and risk of operative delivery [2-4]. There was also no consensus on the time limitation for exposure to extra-amniotic Foley catheter balloon. Some practitioners set a maximum time limit [2-3], while others wait until spontaneous expulsion of the balloon catheter [4-5]. Cromi reported cutting the maximum time for cervical ripening (from 24 to 12 hours) would increase the proportion of women who deliver vaginally within 24 hour after Foley catheter insertion [6].

In our previous research, we have proved that Foley catheter can be safely used in term labor induction. However the vaginal delivery rate within 24 hours is relatively low with an 80mL balloon for a maximum of 24 hours ripening. Our present study, therefore, aims to determine whether different time length of ripening (12 vs. 24 hours) and different balloon size (30 vs. 80 mL) would influence the efficacy of a transcervical Foley catheter used in labor induction.

**2. Aims of the trial**

The objective of this trial is to explore the optimal balloon size and ripening time when Foley catheter is used in term labor induction. Specifically we aim to study:

1. The effect of 2 balloon sizes and 2 ripening time limits on the outcomes of induction when Foley catheter is used.
2. Maternal and neonatal complications in Foley catheter balloon induction.
3. Labor patterns of induced vs. spontaneous labor.

**3. Study Design**

1. **Study Design** It is an open-label, randomized, controlled trial in a multi-center setting. Participants will be randomly allocated to one of four parallel groups, in 1:1:1:1 ratio, to receive either one of four treatments: (1) 30 mL balloon for a maximum of 12 hours, (2) 30 mL balloon for a maximum of 24 hours, (3) 80 mL balloon for a maximum of 12 hours, (4) 80 mL balloon for a maximum of 24 hours. Neither the investigator nor the participants are blind to the assignment.
2. **Randomization** The randomization sequence is computer-generated with variable blocks in 1:1:1:1 allocation for each arm by the statistician (Dr. Biyun Xu). Since 95% of women undergoing labor induction in our district are nulliparous, we will not stratify women according to parity. The randomization sequence will be placed into numbered opaque envelopes by two doctors uninvolved in the trial before the initiation of the study. None of the clinical investigators will have access to the schedule until completion of the study.
3. **Inclusion /Exclusion Criteria** Women scheduled for labor induction will be screened for eligibility. Inclusion criteria are gestational age ≥ 37 weeks; singleton gestation with cephalic presentation; intact membranes; reactive non-stress test and Bishop’s score < 6. Women less than 18 years, with multiple pregnancy, abnormal pelvimetry, abnormal presentation, placenta previa, vaginal infection, intrauterine fetal death, or GBS positive will be excluded.
4. **Primary Outcome** The primary outcome is vaginal delivery within 24 hours.
5. **Secondary outcomes** Secondary maternal outcomes are cesarean section rate, instrumental vaginal delivery, indications for operative delivery, induction-to-delivery time, induction-to-active phase time, epidural analgesia, oxytocin use, uterine hyperstimulation, chorioamnionitis, postpartum hemorrhage (blood loss >500ml within 24h after delivery)，postpartum transfusion, cervical dilation and station. Secondary neonatal outcomes include birth weight, the rate of Apgar scores of less than 7 at 5 minutes and neonatal admission.
6. **Duration of the Project** We will recruit participants between February 2013 and June 2014.
7. **Sample Size** Our previous study using an 80mL balloon with a maximum of 24 hours indicated a vaginal delivery rate in 24 hours of 28.1%[7]. A review of studies of various balloon sizes and ripening time showed a vaginal delivery rate within 24 hours ranges from 48% to 66%. Under this scenario, we assumed that by revising the Foley catheter induction protocol we could achieve a clinically significant increase in vaginal delivery within 24 hours. Thus planned sample size for this investigation was 126 patients each treatment group to show an increase in vaginal delivery rate within 24 hours from 28% to 48% with a two-sided test (α error=5%, power=80%). Formula: $n=2\lambda/{(2\sin^{-1} \sqrt{p_{max}}-2\sin^{-1} \sqrt{p_{min}})}^{2}$
8. **Labor patterns** To investigate the progress of labor in women induced with Foley catheter versus spontaneous labor, we will prospectively collect the information of women with spontaneous onset of labor and vaginal delivery in a 1:1 ratio in Nanjing Drum Tower Hospital. Women will be eligible as control group if they are nulliparous, singleton pregnancy in vertex presentation, less than 2 cm dilated at admission, and have spontaneous onset of labor. We will exclude women who deliver preterm, have fetus with congenital anomalies, or deliver by cesarean section. Participants need to sign written informed consent and clinical data will be used for research. Maternal age will be matched and difference of gestational age at delivery should be less than 7 days.

**4. Settings**

| Institutions | No. Of participants to recruit |
| --- | --- |
| Nanjing Drum Tower Hospital (leader institution) | 296 |
| Wuxi Maternal and Child Health Hospital | 52 |
| Taixing People's Hospital | 52 |
| The First People's Hospital of Kunshan | 52 |
| Jintan People's Hospital | 52 |

**5. Funding**

Funding for this study is provided by National Key Clinical Department of Ministry of Health of China (2011271).

**6. Implementation of the trial**

1. **Implementation methods of Foley catheter balloon induction**

**A. Recruitment and consent**

After admission for term labor induction, women will be evaluated for indications and contraindications for induction by the obstetricians. Gestational age will be confirmed and the status of the cervix will be determined by the Bishop pelvic scoring system [8]. Vaginal discharge will be tested to exclude vaginal infection. Ultrasound results will be reviewed to exclude placenta previa. Potential participants will be informed about the trial and given information when the decision to induce labor is made after admission. Those women who wish to participate and who understand the nature of the trial will be asked to complete and sign two copies of the consent form. If woman dose not wish to participant in the trial, she will receive other method of induction according to each hospital’s protocol.

**B. Randomization**

After informed consent, the obstetrician and another investigator will open the envelope containing the assignment information. At enrollment, the obstetricians will record medical history and indications for induction in *Induction of Labor Checklist* (Appendix 1.).

**C. Interventions** **Protocol for Foley catheter insertion.** The cervix will be exposed with a sterile speculum and cleansed with a povidone-iodine solution. Under direct visualization, a 16-F Foley catheter will be inserted into the endocervical canal. Once the catheter is past the internal os, the catheter balloon will be inflated with sterile saline solution and pulled against the internal os of the cervix. The external end of Foley catheter will be taped with tension to the medial aspect of the woman’s thigh. Non-stress test will be conducted after catheter insertion. Women in group 1 and group 2 will have a 30ml Foley catheter balloon, with a maximum ripening time of 12 hours and 24 hours respectively. Women in group 3 and group 4 will have an 80ml balloon with a maximum of 12 hours and 24 hours exposure respectively.

The following will be indications for early cessation of treatment: (1) failure to insert the Foley catheter ; (2) severe bleeding or discomfort after insertion; (3) either participants or the supervising obstetricians call for the cessation of the trial. The obstetrician will be informed and still collect outcome data from women who have stopped treatments.

In all groups, the Foley catheter will be removed for the following reasons: (1) the time limitation for ripening is reached; (2) spontaneous rupture of membranes has occurred; (3) the balloon is expelled spontaneously; (4) women enter the active phase of labor; or (5) hyperstimulation or fetal distress is suspected.

**D. Induction after Removal of Balloon**

For women who do not enter spontaneous labor during the ripening process, amniotomy will be performed once the catheter is expelled or removed. Women will be given intravenous oxytocin to induce the labor if the contraction was unsatisfactory after 30 minutes of amniotomy (<3 contractions per 10 min). Intravenous oxytocin will be started at an infusion rate of 1 mIU/min. The dose will be increased by 2 mIU/min every 20 minutes until adequate uterine activity is achieved, defined as uterine activity of 200-250 Montevideo units. The maximal infusion rate permitted is 25 mIU/min. Oxytocin will be discontinued once the women is deemed to be in active labor. If amniotomy is failed, the woman will receive oxytocin induction for 6 hours. Then the doctor will try amniotomy again.

**E. Management of Labor**

When a woman is in active labor, maternal heart rate, blood pressure and temperature will be recorded every 6 hours, fetal heart rate and uterine activity will be continuously recorded with fetal heart monitor. The attending doctors will make clinical decision when there is an abnormal fetal heart tracing or failure to progress.

**F. Postpartum Management**

After delivery, participants will be managed according to postpartum routine of each center. Obstetricians will collect clinical information within 48 hours after discharge of the participants and complete *Case Report Form for Balloon Induction* (Appendix 2.). In addition, we will make telephone calls to every woman 30 days after delivery and record neonatal outcomes and late onset complications, such as puerperal infection and puerperal hemorrhage.

**G. Adverse Reactions**

Hyperstimulation is defined as more than 5 uterine contractions in 10 minutes in consecutive 30-minute intervals with or without fetal heart rate changes [9].

Failed induction is identified as failure to progress into the active phase of labor, despite adequate contraction patterns 24 hours after amniotomy.

Failure to progress is diagnosed as unchanged cervical dilation in a 4-hour interval, despite the establishment of well uterine contraction.

Chorioamnionits is diagnosed as maternal fever（≥38°C），accompanied by maternal tachycardia (>100bpm), or uterine fundal tenderness, or fetal tachycardia (>160bpm), or purulent amniotic fluid [10].

Postpartum hemorrhage is defined as blood loss of more than 500 mL within 24 hours of delivery. Blood loss is measured by: 1) collecting and recording of blood in bedpan containers; 2) weighing of materials including soaked sponges and pads on a scale and subtracting the known dry weights of these materials.

1. **Data collection for spontaneous labor**

**A. Recruitment and consent**

Women will be eligible as control group if they are nulliparous, singleton pregnancy in vertex presentation, less than 2 cm dilated at admission, and have spontaneous onset of labor. We will exclude women who deliver preterm, have fetus with congenital anomalies, or deliver by cesarean section. Participants need to sign written informed consent and clinical data will be used for research. Maternal age will be matched and difference of gestational age at delivery should be less than 7 days.

**B. Data collection**

Obstetricians will collect clinical information within 48 hours after discharge of the participants and complete *Case Report Form for Spontaneous Labor* (Appendix 3.). In addition, we will make telephone calls to every woman 30 days after delivery and record neonatal outcomes and late onset complications, such as puerperal infection and puerperal hemorrhage.

1. **Schedule**
2. Preparation phase (From June 2012 to Jan 2013)

In this phase, we have focused on preparations for the trial protocol and meeting with the coordinator of each center. Staff has been trained on Bishop scoring and Foley catheter balloon insertion. Oxytocin induction protocols has been designed and tested. Research protocol and management systems have been put in place.

1. Implementation of the trial (From Feb 2013 to Jun 2014)

Implementation of the randomized controlled trial will start. Pregnant women will be enrolled and followed up until 30 days postpartum. Once the target number of pregnancies has been enrolled, enrollment will be discontinued.

1. Analysis of data and report preparation (From Jul 2014 to Dec 2014)

Scientific papers, reports and presentations of the findings will be made.

**7. Statistical Analysis**

Statistical analysis of outcomes data will be performed with SPSS 17.0. Data will be analyzed on an intention-to-treat basis. Normally distributed data will be presented as means with SDs; skewed distributions will be presented as medians with IQRs or medians with ranges. Categorical outcomes will be summarized using frequency distributions. For quantitative data, ANOVA or rank sum test will be used. For categorical data, we will calculate p values with Chi-square or Fisher exact tests. For time-to-delivery data, we will construct Kaplan-Meier survival curves and calculate log-rank test and p values. A probability value of 0.05 will be used as the cut-point for significance.

**8. Investigators**

| Name | Institution |  |
| --- | --- | --- |
| Yali Hu | Nanjing Drum Tower Hospital | Primary Investigator |
| Lingqing Hu | Wuxi Maternal and Child Health Hospital | Coordinator |
| Biao Xu | Taixing People's Hospital | Coordinator |
| Qin Liu | The First People's Hospital of Kunshan | Coordinator |
| Yi Ding | Jintan People's Hospital | Coordinator |
| Zhiqun Wang | Nanjing Drum Tower Hospital | Coordinator |
| Yimin Dai | Nanjing Drum Tower Hospital | Data collection |
| Ning Gu | Nanjing Drum Tower Hospital | Data collection |
| Biyun Xu | Nanjing Drum Tower Hospital | Statistician |
| Mingming Zheng | Nanjing Drum Tower Hospital | Data collection |
| Jingxian Ling | Nanjing Drum Tower Hospital | Data collection |
| Lei Zhang | Nanjing Drum Tower Hospital | Data collection |
| Lili Qiu | Nanjing Drum Tower Hospital | Auditor |
| Qiao Weng | Nanjing Drum Tower Hospital | Data manager |

**9. Management of adverse events**

1. Hyperstimulation

Foley catheter balloon should be removed if it is in situ and the patient should take a lateral position. Tocolysis also can be used in an attempt to correct uterine tachysystole. If a Category III FHR tracing occurs and there is no response to routine corrective measures cesarean delivery should be considered.

1. Abnormal fetal heart monitoring

Management of abnormal fetal heart tracing includes provision of maternal oxygen, change in maternal position, discontinuation of labor stimulation, treatment of maternal hypotension, and treatment of tachysystole with FHR changes. If a Category III tracing does not resolve with these measures and assisted vaginal delivery or cesarean delivery should be considered.

1. Chorioamnionitis

Broad spectrum antibiotics for treatment of intrapartum infection will be administered when maternal temperature is ≥38 degrees C. A first line antibiotic is cefoxitin 2g, q6h. If a woman is allergic to cefoxitin, clindamycin 0.9g, q8h can be use.

**10. Data management**

Once a participant has agreed to join the trial, the obstetrician will record demographic and obstetrical data on CRF (case report form). CRF contains no patient private information (name, address, or telephone number). Data manager will enter the data into database. If the data is incorrect or missing, data manager will report to the auditor and investigator. The original case note will be accessed and the data required for the CRF will be extracted. The statistician will complete the analysis of the data.

**References**

1. Jozwiak M, Bloemenkamp KW, Kelly AJ, et al. Mechanical methods for induction of labour Cochrane Database Syst Rev. 2012, 14; 3:CD001233.
2. Pennell CE, Henderson JJ, O’Neill MJ, et al. Induction of labour in nulliparous women with an unfavourable cervix: a randomised controlled trial comparing double and single balloon catheters and PGE2 gel. BJOG, 2009; 116: 1443–52.
3. Levy R, Kanengiser B, Furman B, et al. A randomized trial comparing a 30-mL and an 80-mL Foley catheter balloon for preinduction cervical ripening. Obstet Gynecol 2004; 191(5):1632–6.
4. Delaney S, Shaffer B, Cheng Y, et al. Labor Induction With a Foley Balloon Inflated to 30 mL Compared With 60 mL. Obstet Gynecol 2010; 115:1239-1245.
5. [Prager M](http://www.ncbi.nlm.nih.gov/pubmed?term=Prager%20M%5BAuthor%5D&cauthor=true&cauthor_uid=18715244), [Eneroth-Grimfors E](http://www.ncbi.nlm.nih.gov/pubmed?term=Eneroth-Grimfors%20E%5BAuthor%5D&cauthor=true&cauthor_uid=18715244), [Edlund M](http://www.ncbi.nlm.nih.gov/pubmed?term=Edlund%20M%5BAuthor%5D&cauthor=true&cauthor_uid=18715244), [Marions L](http://www.ncbi.nlm.nih.gov/pubmed?term=Marions%20L%5BAuthor%5D&cauthor=true&cauthor_uid=18715244). A randomised controlled trial of intravaginal dinoprostone, intravaginal misoprostol and transcervical balloon catheter for labour induction. BJOG. 2008;115(11):1443-50.
6. Cromi A, Ghezzi F, Agosti M, et al. Is transcervical Foley catheter actually slower than prostaglandins in ripening the cervix? A randomized study. Am J Obstet Gynecol 2011; 204:338.e1-7.
7. Zheng MM, Hu YL, Zhang SM, Ling JX, Wang ZQ. Trans-cervical Foley catheter balloon versus vaginal prostaglandin E2 suppository for cervical ripening and induction of labor: a prospective randomized controlled trial. Chinese Journal of Perinatal Medicine. 2011; 14: 648-652.
8. Bishop EH. Pelvic scoring for elective induction. Obstet Gynecol 1964; 24:266–8.
9. ACOG Committee. [ACOG Practice Bulletin No. 107: Induction of labor.](http://www.ncbi.nlm.nih.gov/pubmed/19623003) Obstet Gynecol. 2009; 114:386-97.
10. [Hauth JC](http://www.ncbi.nlm.nih.gov/pubmed?term=Hauth%20JC%5BAuthor%5D&cauthor=true&cauthor_uid=4011072), Gilstrap LC 3^rd^, Hankins GD, et al. Term maternal and neonatal complications of acute chorioamnionitis.Obstet Gynecol. 1985;66(1):59-62.

**Appendix 1. Induction of Labor Checklist**

| Date | Age |  |
| --- | --- | --- |
| Patient ID | G/P | EDC |
| Gestational age | Indication for induction |  |

- Confirmation of term gestation
  - An ultrasound prior to 20 weeks confirms gestation of at least 39 complete weeks
  - Fetal heart tones have been documented
- If induction is indicated before 39 weeks, senior doctors should be consulted before induction.
- Cephalic presentation
- Intact membrane
- Adequate pelvis
- Normal placenta
- Normal vaginal discharge
- Reactive NST
- Estimated fetal weight
- GBS status
- Medical history and Lab results
  - Allergies
  - Medical complications
  - Obstetric complications
  - Lab results（blood routine, blood type, electrocardiogram,etc.）
- Informed consent
- Bishop score
- Intevention Group

| - - 30mL balloon×12h | - - 80mL balloon×12h |
| --- | --- |
| - - 30mL balloon×24h | - - 80mL balloon×24h |

Physician Signature

**Appendix 2. Case Report Form for Balloon Induction**

| Year of Birth | (m/year) | | | Patient ID | | | |  |
| --- | --- | --- | --- | --- | --- | --- | --- | --- |
| Height | cm | | | Wt before Delivery | | | | Kg |
| GA | w d | | | G/P | | | |  |
| Indication | - □Prolonged gestation - □FGR - □Medical | | | - □GDM／DM   □Oligohydramnion | | | | - □PIH   □ICP  □Other |
| Group | □ 30mL Balloon×12h  □ 80mL Balloon×12h | | | □30mL Balloon×24h  □80mL Balloon×24h | | | |  |
| Time of induction |  | | | Bishop before induction | | | |  |
| Time of Removal |  | | | Bishop after induction | | | |  |
| Indication for | □Spontaneous | | | □Time limit reached | | | | □Abnormal CTG |
| Balloon Removal | □Hyperstimulation | | | □Rupture of membrane | | | | □Abnormal Bleeding |
|  | □Active labor | | | □Discomfort | | | |  |
| Induction after | □Amiotomy | | | Time | | | |  |
| Balloon Removal | □Oxy  □Max dose of Oxy | | | Time | | | |  |
|  | □Other | | | Time | | | |  |
| Labor patterns |  | | |  | | | |  |
| Time | | | Dilation of cervix | | | Decadence of presentation | | |
|  | | |  | | |  | | |
|  | | |  | | |  | | |
|  | | |  | | |  | | |
|  | | |  | | |  | | |
|  | | |  | | |  | | |
|  | | |  | | |  | | |
|  | | |  | | |  | | |
|  | | |  | | |  | | |
|  | | |  | | |  | | |
|  | | |  | | |  | | |
|  | | |  | | |  | | |
|  | | |  | | |  | | |
| Time of Delivery | |  | | |  | |  | |
| Mode of Delivery | | □Vaginal Spontaneous | | | □CS | | □Vaginal Instumental | |
| Indication for CS | | □Failed indution | | | □Abnoral Labor | | □Other | |
|  | | □Abnormal CTG | | | | | | |
| Indication for Assisted | | Vaginal Delivery | | |  | |  | |
| Analgesia | | □Epidural | | |  | |  | |
| PPH | | ml | | | Blood Transfusion | | □ Yes □No | |
| Maternal infection | | □No | | | □Chorioamnionitis | | □Endometritis | |
| Hyperstimulation | | □No □Yes | | | | |  | |
|  | | □ Uterus Rupture | | |  | | □ Placenta Abruption | |
| Other complications  Postpartum Diagnosis | |  | | | Postpartum Stay | | D | |
| Neonatal Birth Wt | |  | | | Apgar Score | | □1min 5min | |
| Neonatal Admission | | □Ward □NICU | | | □No | |  | |
| Indication | | □Infection | | | □Asphyxia | | □MAS | |
|  | | □RDS | | | □Low birth weight | | □HIE | |
|  | | □Other | | | □Neonatal Death | |  | |

Duration of Stay D

**Appendix 3. Case Report Form for Spontaneous Labor**

| Patient ID |  | Nulliparous | □Yes |  |  |
| --- | --- | --- | --- | --- | --- |
| Age（y） |  | Height（cm） |  | Weight（kg） |  |
| Time of Admission |  | Complications |  |  |  |
| Dilation at Admission |  | Prsentation |  |  |  |
| Oxy augmentation | □Yes | Duration (h) |  | MaxOxy Dose | mIU/mL |
| Epidural analgesia | □Yes | □ No |  | PPH |  |
| Gestational Age |  | Neonatal Wt |  |  |  |
| Neonatal Admission | □Yes | □ No | Blood | Transfusion | □Yes □ No |
| Assisted Vaginal Delivery | □Yes | Indication |  |  |  |

**Labor patterns**

| Time | Dilation of cervix | Decadence of presentation |
| --- | --- | --- |
|  |  |  |
|  |  |  |
|  |  |  |
|  |  |  |
|  |  |  |
|  |  |  |
|  |  |  |
|  |  |  |
|  |  |  |
|  |  |  |
|  |  |  |
